# Serum Fatty Acids and Risk of Cutaneous Melanoma: A Population-Based Case-Control Study

**DOI:** 10.1155/2013/659394

**Published:** 2013-01-28

**Authors:** Marco Vinceti, Carlotta Malagoli, Laura Iacuzio, Catherine M. Crespi, Sabina Sieri, Vittorio Krogh, Sandra Marmiroli, Giovanni Pellacani, Elisabetta Venturelli

**Affiliations:** ^1^Departments of Diagnostic, Clinical and Public Health Medicine, Environmental, Genetic and Nutritional Epidemiology Research Center (CREAGEN), University of Modena and Reggio Emilia, Padiglione De Sanctis, Via Amendola 2, 42122 Reggio Emilia, Italy; ^2^Department of Biostatistics, University of California Los Angeles Fielding School of Public Health, Los Angeles, CA 90095-1772, USA; ^3^Epidemiology and Prevention Unit, National Cancer Institute, Via Venezian 1, 20133 Milan, Italy; ^4^Department of Surgery, Medicine, Odontoiatrics and Morphological Sciences, University of Modena and Reggio Emilia, Via del Pozzo 71, 41124 Modena, Italy

## Abstract

*Background*. Some observational studies have suggested that excess dietary intake of polyunsaturated fatty acids such as linoleic acid increases cutaneous melanoma risk. We aimed at examining the association between serum fatty acids and melanoma risk by conducting a population-based case-control study in a northern Italy community. *Methods*. The percentage composition of 12 fatty acids was determined in 51 newly diagnosed melanoma patients and 51 age- and sex-matched population controls by extracting total lipids from serum samples using thin layer and gas chromatography. Conditional logistic regression was used to estimate the relative risk of melanoma associated with tertiles of percentage composition of each fatty acid as well as groupings including saturated, monounsaturated, and polyunsaturated fatty acids. *Results*. We found a slightly increased melanoma risk for stearic and arachidic acids proportion, with and without adjustment for potential confounders. For an n-3 polyunsaturated fatty acid, docosapentaenoic acid, we found a male-specific direct association with melanoma risk. No other associations emerged for the other saturated, monounsaturated, and polyunsaturated fatty acids, individually or grouped by type. *Conclusions*. These findings do not suggest a major role of fatty acids, including linoleic acid, on risk of cutaneous melanoma, though their evaluation is limited by the small sample size.

## 1. Introduction

A steady increase of melanoma incidence rates has been observed in recent decades in many countries [1–3]. A report from England on trends in cancer occurrence in 1991–2000 ranks melanoma incidence as the most rapidly increasing among all site-specific neoplasms, and early detection due to screening does not appear to be the only explanation of the growing incidence of the disease [4].

This points to a role of environmental risk factors in increasing melanoma incidence, but the exact nature of these factors, apart from ultraviolet exposure, is still unclear [5]. In particular diet, a potentially modifiable risk factor, has long been suspected to play a role in melanoma occurrence, though the results of epidemiologic investigations carried out so far have yielded inconsistent results [6–13]. High intake of polyunsaturated fatty acids (PUFAs), and of linoleic acid in particular, have been linked to increased melanoma risk [6, 10, 14]. In contrast, high intake of fat [15] and of fish rich in n-3 fatty acids [12, 16] has been associated with reduced risk of the disease. Laboratory studies have reported toxic effects of fatty acids such as linoleic and arachidonic acid on distinct cell types such as leukemia cells [17], rabbit renal cells [18], human endothelial cells [19], and murine and human melanoma cell lines [20], with different potential mechanisms including *in vivo* isomerization into *trans* fatty acids [21]. In epidermal reconstructs, n-6 PUFAs administration resulted in increased epidermal oxidative damage, possibly leading to accumulated mutations and increased risk of skin cancer, especially melanoma [22].

Taking into account these findings and the scarcity of human studies, in the present investigation we explored the association between serum fatty acid distribution and the risk of cutaneous melanoma in a population-based case-control study.

## 2. Methods

### 2.1. Study Population

We invited the patients newly diagnosed with cutaneous melanoma attending the Dermatologic Clinic of Modena University Hospital in the period 1999–2002 to participate in a population-based case-control study. Inclusion criteria were residence in Modena Province, an area in northern Italy with approximately 700,000 inhabitants, and a histologically confirmed diagnosis of cutaneous melanoma without clinical evidence of metastasis. Fifty-nine of 84 eligible patients (72.0%) agreed to participate in the study. We randomly selected a resident of Modena Province, sex- and age-matched (±5 years) to each patient, from a database available at the Modena Local Health Authority. Eligible controls were contacted by telephone and enrolled in the study after obtaining their informed consent. The participation rate among eligible population controls was 56.3%. 

### 2.2. Data Collection

We asked participants to go, early morning and fasting, to the Dermatologic Clinic of Modena where they provided a 10 cc blood sample and completed a questionnaire on demographic characteristics and lifestyle habits, including indicators of sun exposure and diet using the EPIC semiquantitative food frequency questionnaire [10, 13]. Blood specimens were collected in EDTA tubes, immediately separated and stored at −20°C until analysis. Subjects whose blood samples were separated or stored more than 24 hours after collection and whose samples were not available for other reasons were excluded from analyses, leaving 51 cases and their matched controls. A dermatologist determined each participant's phototype using the Fitzpatrick phototyping scale [22] and collected information about family history of melanoma, sun exposure, and other anamnestic characteristics.

### 2.3. Fatty Acid Determination

We determined serum fatty acid percentage composition of the following twelve fatty acids: 16 : 0 (palmitic acid), 17 : 0 (margaric acid), 18 : 0 (stearic acid), 18 : 1n-9 (oleic acid), 18 : 2n-6 (linoleic acid), 20 : 0 (arachidic acid), 20 : 4n-6 (arachidonic acid), 20 : 5n-3 (eicosapentaenoic acid, EPA), 22 : 5n-3 (7,10,13,16,19-docosapentaenoic acid, DPA), and 22 : 6n-3 (4,7,10,13,16,19-docosahexaenoic acid, DHA), 24 : 0 (lignoceric acid), and 24 : 1n-9 (nervonic acid). We also calculated the serum proportion of the following four fatty acid categories: SFAs—saturated fatty acids (palmitic acid, margaric acid, stearic acid, arachidic acid, lignoceric acid), MUFAs—monounsaturated fatty acids (oleic acid, nervonic acid); n-6 PUFAs—polyunsaturated fatty acids (linoleic acid, arachidonic acid); n-3 PUFAs (EPA, DPA, DHA). Fatty acid proportion was determined in serum phospholipids by extracting total lipids from serum samples using thin layer chromatography (TLC) and gas-chromatography (GC). Menhaden oil purchased from Supelco; Bellefonte, PA, and phosphatidylcholine (LIPOSTABIL vials 250 mg/5 mL; A. Notterman, Colonia, Germany; distributed by Aventis-Pharma, Milan) were used as standards. Total lipids were extracted from serum samples (400 *μ*L) with 2 mL of chloroform-methanol 2 : 1 (v : v) (35). After centrifugation, the organic layer was transferred into a tube and evaporated under nitrogen. The dried residue was dissolved in 60 *μ*L of chloroform-methanol 2 : 1 (v : v) and 50 *μ*L of the obtained sample was purified by TLC with a development mobile-phase composed by A : B (70 : 30 v : v) where A = chloroform and B = methanol : water : ammonia 93 : 5 : 2 (v : v : v). Phospholipidic fraction was identified to spray phosphatidylcholine standard with a solution of phosphomolibdic acid 10% in methanol. Phospholipidic fraction of samples was extracted, twice, with 2 mL of chloroform-methanol 2 : 1 (v : v), and fatty acid methyl esters (FAME) of the phospholipidic class were obtained through transesterification with 2 mL of hydrochloric acid in methanol (3 N) (Supelco, Bellefonte, PA). FAME were further extracted into 1 mL of hexane and 0.5 *μ*L of the mixture was injected for gas-chromatographic measurement.

The GC analysis was performed using an 8610 DANI instrument (Monza, Italy) equipped with a temperature vaporizer injector and a flame ionization detector. We introduced the samples into the capillary column using a split-splitless technique, using a BPX70 column (30 m × 0.25 mm i.d.) with 0.25 *μ*m film thickness purchased from SGE (Ringwood, Australia). The gas conditions were: carrier gas helium at flow rate of 0.8 mL/min; hydrogen 0.5 mL/min; make-up gas nitrogen 0.5 mL/min. The oven temperature increased from 130°C to 210°C at a rate of 3°C/min, from 210°C to 260°C at a rate of 5°C/min, then kept constant for 3 minutes. Identification of FAME was obtained by comparison with the relative retention times of a commercial standard solution (Menhaden oil). The chromatograms were recorded and processed by computer-aided methods. The relative amount of each FAME (percent of total area) was quantified by integrating the peak and dividing the results by total area for all fatty acids identified. The detection limit was 0.01% for all fatty acids. Within-day coefficients of variation (CV) were based on the analysis of the same serum sample, extracted and analyzed 10 times during the same day. CV ranged from 0.6% for large peaks to 38.6% for small peaks. The between-day CVs were based on the analysis of one serum sample on separate days (up to 19 days). CV for all fatty acids were: 5.20% for palmitic, 28.15% for margaric, 6.20% for stearic, 69.31% for arachidic, 39.12% for lignoceric, 7.65% for oleic, 47.21% for nervonic, 6.55% for linoleic, 9.82% for arachidonic, 23.01% for EPA, 24.31% for DPA, 18.89% for DHA. Serum samples were analysed in 19 consecutive cycles; for every cycle we added a standard (cod liver oil) and a quality control serum sample. Data were expressed as percentage of each fatty acid calculated on the total amount of fatty acids separated from the seric phospholipidic fraction. Determinations below the detection limit (0.01%) were assigned a value of 0.01%.

### 2.4. Statistical Analysis

We computed odds ratios (OR) as an estimate of relative risk according to categories of fatty acid serum levels, based on a matched analysis using conditional logistic regression. For these analyses, levels of serum fatty acids were categorized into tertiles based on the distribution in controls, using the bottom tertile as the reference group. We also tested for linear trend by entering fatty acid composition as continuous variable. We repeated analyses adjusting for phototype, sun exposure, and education in multivariate analysis, and conducted sex- and age- (≥50 versus <50 years) stratified analyses. We also compared mean dietary intakes of selected fatty acids and fatty acids categories using Student's *t*-test.

## 3. Results

Demographic and clinical characteristics of cases and controls are reported in Table 1. Cases were similar to controls on educational attainment, had somewhat lower phototypes, and reported a higher occurrence of sunburns than controls.

Table 2 summarizes the fatty acid distribution in sera of study participants, and Table 3 reports the ORs for cutaneous melanoma according to percentage composition of each serum fatty acid in unadjusted analyses. The proportions of stearic and arachidic acid were directly associated with increased melanoma risk, though these associations were limited and statistically imprecise. Compared with the lowest tertile, ORs for the highest tertile were 1.94 (95% CI, 0.61–6.20; *P* trend = 0.115) for stearic acid and 2.13 (95% CI, 0.61–7.46; *P* trend = 0.400) for arachidic acid. However, no association was detected for the overall SFAs group. No association was found with any of the other unsaturated fatty acids or with their groupings, nor with the ratios between the fatty acid categories.

We also compared cases and controls with respect to distribution of dietary intakes of the fatty acids which could be estimated with the EPIC food frequency questionnaire (saturated fatty acids, monounsaturated and polyunsaturated fatty acids, oleic, linoleic and linolenic acid). Mean intakes were similar in the two groups, with the exception of a somewhat higher linoleic acid intake in cases (8.48 g/day) compared with controls (7.75 g/day, *P* = 0.242). 

ORs adjusted for educational attainment, skin phototype, and sun exposure are shown in Table 4. An increased risk of melanoma was confirmed for stearic acid (OR for the highest tertile 3.00, 95%CI 0.56–16.07, *P* trend 0.101) and arachidic acid (OR for the highest tertile 2.05, 95% CI 0.45–9.40, *P* trend 0.334). A weak and statistically very unstable indication of an inverse association between melanoma risk and linoleic fatty acid also emerged (OR for the highest tertile 0.71, 95% CI 0.20–2.45, *P* trend 0.420). We performed a further multivariate analysis also adjusting for estimated dietary intakes of vitamin D, fruit and vegetables: none of these variables substantially modified the results.

When we stratified on sex and age, there was little indication of any interaction with these factors. The only notable finding was for the sex-specific analysis for docosapentaenoic acid, for which we observed a strong male-specific positive association with melanoma risk (OR 32.24 for the highest tertile, 95%CI 1.68–616.34, *P* trend 0.029), that was confirmed in multivariate analysis.

## 4. Discussion

A direct relation between melanoma risk and dietary intake of fatty acids, particularly PUFAs, has been observed or suggested in some studies [6, 10, 14, 23], though other epidemiologic investigations did not detect this association [24–27] or found an inverse association [16]. However, only one study on this topic using biomarkers (percentage of fatty acids in subcutaneous adipose tissue) has been performed to date [14], to the best of our knowledge, and therefore the present one appears to be the first study on fatty acids as a risk factor for melanoma analyzing serum distribution rather than assessing their dietary intakes from food questionnaires. 

We found positive associations between melanoma incidence and serum proportions of stearic and arachidic acid. This finding has some consistencies with the results of the case-control study by Millen et al. [9], who reported an increased melanoma risk for higher intakes of saturated fat. In contrast, our previous studies based on assessment of dietary intake did not suggest such association [10, 13]. For PUFAs and linoleic acid, in the present investigation we observed no association and a weak inverse association with risk, respectively, in contrast with our previous observations in this study population of an increased melanoma risk associated with n-6 PUFAs levels and with linoleic acid itself [10, 13], and with the excess melanoma risk among high consumers of cod liver oil, a supplement rich in n-3 PUFA, in a Norwegian cohort [6]. In sex-specific analyses, the n-3 PUFA docosapentaenoic acid was positively associated with melanoma risk in males, a result inconsistent with the observation by Veierod et al. of an increased melanoma risk associated with use of PUFA intake in females and not in males [6]. We speculate that the inconsistencies of the results between investigations based on dietary intake and the present study might be due to methodological issues such as the different indicator of fatty acid intake adopted (dietary intake of fatty acids instead of serum levels/percentage composition as in the present investigation), or by uncontrolled confounding. The inconsistencies may also be due to chance.

Strengths of the present study include its population-based design, which reduces the risk of selection bias compared with hospital-based studies [28], and the adjustment for potential confounders, such as phototype, education, and sun exposure, known or suspected to influence melanoma risk. This study also has important limitations: its small sample size, which led to low statistical precision and hampered the detection of possible minor effects on risk; the possibility of residual confounding; and the use of percentage composition of serum fatty acids instead of their absolute levels, an approach which might have hidden some associations compared with an analysis based on serum concentrations of the fatty acids. In addition, the fatty acid assays were conducted postdiagnosis, and may not reflect the relevant prediagnosis composition.

In conclusion, taken as a whole, this study yielded little evidence of an association between melanoma risk and circulating fatty acids. However, there was limited evidence for a direct relation between melanoma risk and stearic and arachidic acids, and docosapentaenoic acid in males, which may be worth further investigation.

## Figures and Tables

**Table 1:** Characteristics of study subjects.

	Cases		Controls	
Characteristic	*n*	(%)	*n*	(%)
	51	(100)	51	(100)
Sex				
Male	23	(44.1)	23	(44.1)
Female	28	(54.9)	28	(54.9)
Age				
25–49 y	17	(33.3)	16	(31.4)
50–79 y	34	(66.7)	35	(68.6)
Educational attainment level				
Elementary school or less	18	(35.3)	17	(33.3)
Middle school	8	(15.7)	8	(15.7)
High school	17	(33.3)	17	(33.3)
College or more	8	(15.7)	9	(17.7)
Phototype				
I	3	(5.9)	1	(2.0)
II	25	(49.0)	16	(31.4)
III	21	(41.2)	29	(56.8)
IV	0	(0.0)	5	(9.8)
Missing	2	(3.9)	0	(0)
Sun exposure (days per year)				
0	6	(11.8)	3	(5.9)
<15	23	(45.1)	10	(19.6)
15–30	16	(31.4)	27	(52.9)
>30	5	(9.7)	11	(21.6)
Missing	1	(2)	0	(0)
Life-time history of sunburns				
0	21	(41.2)	33	(64.7)
1	24	(47.1)	14	(27.5)
2	2	(3.9)	2	(3.9)
>2	2	(3.9)	2	(3.9)
Missing	2	(3.9)	0	(0)

**Table 2:** Fatty acid plasma percentage distribution in study subjects.

Systematic name	Common name	Short designation	Cases		Controls			
			Min	Max	Min	Max	33th centile^a^	67th centile^a^
SFAs^b^								
Hexadecanoic	Palmitic	(16 : 0)	27.70	42.75	26.35	42.75	35.10	36.92
Heptadecanoic	Margaric (daturic)	(17 : 0)	0.19	3.20	0.01	4.97	0.52	0.64
Octadecanoic	Stearic	(18 : 0)	11.84	22.41	8.52	21.89	16.53	18.10
Eicosanoic	Arachidic	(20 : 0)	0.01	1.11	0.01	1.26	0.32	0.47
Tetracosanoic	Lignoceric	(24 : 0)	0.01	2.25	0.01	1.65	0.72	1.11
MUFAs^b^								
cis-9-octadecenoic	Oleic	(18 : 1n-9)	9.52	41.69	8.21	38.47	12.72	14.88
cis-15-tetracosenoic	Nervonic	(24 : 1n-9)	0.01	4.02	0.01	3.52	1.04	1.62
PUFAs^b^								
9,12-octadecadienoic	Linoleic	(18 : 2n-6)	11.34	23.56	11.32	23.54	17.19	20.07
5,8,11,14-eicosatetraenoic	Arachidonic	(20 : 4n-6)	0.28	7.41	1.08	6.77	2.75	3.77
5,8,11,14,17-eicosapentaenoic	EPA	(20 : 5n-3)	0.01	1.44	0.01	2.35	0.14	0.33
7,10,13,16,19-docosapentaenoic	DPA	(22 : 5n-3)	0.01	0.60	0.01	0.75	0.09	0.18
4,7,10,13,16,19-docosahexaenoic	DHA	(22 : 6n-3)	0.18	2.06	0.01	1.95	0.43	0.66

			Mean	Standard deviation	Mean	Standard deviation	33th centile^a^	66th centile^a^

Groupings								
SFAs			55.95	3.59	55.68	4.12	54.57	56.72
MUFAs + PUFAs			39.29	3.86	39.37	4.18	38.34	40.73
MUFAs			16.27	5.02	16.22	4.99	14.31	16.52
PUFAs			23.02	4.59	23.15	3.60	21.58	25.06
SFAs/MUFAs + PUFAs			1.44	0.21	1.44	0.26	1.35	1.48
SFAs/PUFAs			2.54	0.58	2.47	0.47	2.23	2.61
n-3 PUFAs			1.10	0.60	1.06	0.69	0.71	1.11
n-6 PUFAs			21.92	4.33	22.09	3.40	20.53	24.04
n-3 PUFAs/n-6 PUFAs			0.05	0.02	0.05	0.03	0.03	0.05

^a^Percentile cutpoints based on fatty acid distribution in controls.

^b^SFAs: saturated fatty acids; MUFAs: monounsaturated fatty acids; PUFAs: polyunsaturated fatty acids.

**Table 3:** Odds ratio (OR) with 95% confidence intervals (CI) of melanoma by tertile of serum fatty acid percentage composition.

	I^a^	II		III		*P* trend
	OR	OR	(95% CI)	OR	(95% CI)
SFAs^b^						
Palmitic	1.00	1.00	(0.37–2.71)	0.75	(0.23–2.41)	0.568
Margaric (daturic)	1.00	0.85	(0.30–2.47)	1.29	(0.40–4.21)	0.783
Stearic	1.00	1.48	(0.50–4.42)	1.94	(0.61–6.20)	0.115
Arachidic	1.00	1.76	(0.44–7.02)	2.13	(0.61–7.46)	0.400
Lignoceric	1.00	0.69	(0.24–1.99)	0.57	(0.18–1.77)	0.725
MUFAs^b^						
Oleic	1.00	1.02	(0.42–2.46)	1.23	(0.44–3.45)	0.909
Nervonic	1.00	1.79	(0.51–6.24)	1.59	(0.32–7.94)	0.876
PUFAs^b^						
Linoleic	1.00	0.73	(0.27–1.98)	0.77	(0.28–2.15)	0.406
Arachidonic	1.00	0.57	(0.21–1.55)	1.33	(0.53–3.33)	0.402
EPA	1.00	2.17	(0.68–6.90)	1.16	(0.30–4.43)	0.600
DPA	1.00	1.2	(0.44–3.24)	1.00	(0.34–2.91)	1.000
DHA	1.00	0.65	(0.22–1.90)	0.67	(0.24–1.86)	0.891
Groupings						
SFAs	1.00	0.41	(0.11–1.48)	1.65	(0.49–5.51)	0.589
MUFAs + PUFAs	1.00	0.81	(0.31–2.16)	0.76	(0.23–2.55)	0.884
MUFAs	1.00	0.95	(0.35–2.59)	1.08	(0.36–3.24)	0.932
PUFAs	1.00	0.57	(0.20–1.64)	0.78	(0.27–2.21)	0.859
SFAs/MUFAs + PUFAs	1.00	1.03	(0.33–3.25)	1.25	(0.41–3.81)	0.907
SFAs/PUFAs	1.00	0.71	(0.26–1.95)	1.33	(0.50–3.46)	0.478
n-3 PUFAs	1.00	0.82	(0.24–2.71)	1.11	(0.37–3.36)	0.718
n-6 PUFAs	1.00	0.61	(0.21–1.74)	0.90	(0.33–2.47)	0.807
n-3 PUFAs/n-6 PUFAs	1.00	1.99	(0.73–5.26)	1.31	(0.42–4.02)	0.679

^a^Referent group.

^b^SFAs: saturated fatty acids; MUFAs: monounsaturated fatty acids; PUFAs: polyunsaturated fatty acids.

**Table 4:** Odds ratio (OR) with 95% confidence intervals (CI) of melanoma by tertile of serum fatty acids percentage composition, adjusted for phototype, education and sun exposure.

	I^a^	II		III		*P* trend
	OR	OR	(95% CI)	OR	(95% CI)
SFAs^b^						
Palmitic	1.00	0.79	(0.22–2.87)	0.71	(0.18–2.76)	0.440
Margaric (daturic)	1.00	0.49	(0.12–1.91)	1.66	(0.39–7.16)	0.646
Stearic	1.00	1.85	(0.46–7.43)	3.00	(0.56–16.07)	0.101
Arachidic	1.00	1.13	(0.20–6.54)	2.05	(0.45–9.40)	0.334
Lignoceric	1.00	0.24	(0.05–1.11)	0.38	(0.09–1.55)	0.552
MUFAs^b^						
Oleic	1.00	1.98	(0.51–7.60)	0.82	(0.22–3.08)	0.977
Nervonic	1.00	1.82	(0.31–10.78)	1.26	(0.15–10.48)	0.305
PUFAs^b^						
Linoleic	1.00	1.10	(0.34–3.58)	0.71	(0.20–2.45)	0.420
Arachidonic	1.00	0.50	(0.14–1.86)	1.10	(0.33–3.71)	0.459
EPA	1.00	1.65	(0.40–6.69)	0.64	(0.12–3.40)	0.785
DPA	1.00	0.49	(0.13–1.85)	0.73	(0.20–2.67)	0.697
DHA	1.00	0.42	(0.09–2.00)	0.44	(0.11–1.74)	0.581
Groupings						
SFAs	1.00	0.28	(0.04–1.73)	1.56	(0.37–6.61)	0.848
MUFAs + PUFAs	1.00	0.62	(0.17–2.26)	0.52	(0.11–2.34)	0.686
MUFAs	1.00	1.33	(0.33–5.29)	0.71	(0.17–2.96)	0.876
PUFAs	1.00	0.95	(0.25–3.59)	0.98	(0.28–3.46)	0.850
SFAs/MUFAs + PUFAs	1.00	1.41	(0.33–6.05)	1.65	(0.43–6.33)	0.962
SFAs/PUFAs	1.00	0.88	(0.25–3.12)	1.31	(0.41–4.14)	0.692
n-3 PUFAs	1.00	0.70	(0.16–3.08)	0.95	(0.23–3.93)	0.531
n-6 PUFAs	1.00	0.64	(0.17–2.46)	0.97	(0.29–3.17)	0.775
n-3 PUFAs/n-6 PUFAs	1.00	1.55	(0.46–5.27)	1.04	(0.25–4.37)	0.504

^a^Referent group.

^b^SFAs: saturated fatty acids; MUFAs: monounsaturated fatty acids; PUFAs: polyunsaturated fatty acids.
